# Mixed Methods in Decision-Making Through Polar Coordinate Technique: Differences by Gender on Beach Handball Specialist

**DOI:** 10.3389/fpsyg.2019.01627

**Published:** 2019-07-12

**Authors:** Juan A. Vázquez-Diz, Juan P. Morillo-Baro, Rafael E. Reigal, Verónica Morales-Sánchez, Antonio Hernández-Mendo

**Affiliations:** ^1^University of Málaga, Málaga, Spain; ^2^Departamento Psicología Social, Trabajo Social, Antropología Social y Estudios de Asia Oriental, Universidad de Málaga, Málaga, Spain

**Keywords:** mixed methods, systematic observation, decision making, polar coordinates, beach handball

## Abstract

The aim of this research was to analyze decision making of the specialist in beach handball in the framework of mixed methods, and through the observation of their actions. To do this, and distinguishing it by genre, an analysis of polar coordinates was realized using the assist and completion of this specific position as behavior criteria. A total of 24 sessions were observed with the HOISAN computer software, using an *ad hoc* design tool. Observation design used was nomothetic, punctual, and multidimensional. The obtained results showed significant relations between the behavior criteria and conditional behaviors (pairing), showing significant relations different for male and female categories. Significant differences were estimated statistically about the importance of the specialist in the development of the static attack in beach handball and their influence in the final result of the match, based on their greater or lesser success in decision making. In the male category it is highlighted that the specialist’s assists are not positively related to a favorable score and that the attack usually ends up in the central areas against closed defensive systems; in addition, their successful completion decisions are produced by means of launches in the central zone, also against closed defensive systems; however, in the female category, specialists’ assists lead to end the attacks in flight on the far right against open defensive systems.

## Introduction

The available literature has brought to light how in collective sports there has been a progressive increase in the physical, technical and tactical demands of competition in recent decades (Carling, 2011; Sarmento et al., 2017). This has generated the necessity of increasing the specific knowledge about these aspects to be able to plan training sessions properly and make a better preparation of the team (Nunes et al., 2015).

Beach handball is not unaware of this situation, as shown by the evolution developed in the modality in all aspects of the game since its inception in the 1990s to the present (Morillo-Baro and Hernández-Mendo, 2015), generating the need for continuous updating and innovation in the professionals of this sport. In the last years, it has undergone a very significant increase, due to the investment that the corresponding sports institutions have made in this sport: European handball federation (EHF), international handball federation (IHF) and the respective national federations developing championships at the state, continental and world level, both of clubs and of the respective national teams (Zapardiel Cortés, 2015).

This modality presents substantial differences with the handball court, both at the regulatory level and in the different facets of the game, turning it into a very new modality, which has successfully broken into the national and international scene. Its own rules of the game make it very attractive, uncertain and interesting (Belančić, 2005). One of the main differences is the way of scoring depending on the different actions: one-point goals can achieve by a classic launch from any player other than the goalkeeper-specialist, or two-point goals can be scored by spin shot of 360-degree, flight shots or by the specialist’s shots (Ostoić and Ohnjec, 2015).

The situation of numerical superiority of the opposing team by means of the defense-attack change of the goalkeeper by the specialist gives him a decisive role in the creation of the offensive game (Gruić et al., 2011). When the game is always developed in offensive numerical superiority, and the possibility of this player to score a two-point goal through a classic shot, makes the player a focus of attention from the defenders of the opposing team. This aspect generates spaces that can be used by teammates through an individual action or an assist. These tactical considerations turn the position of the specialist into one of greater responsibility in decision-making, as the analysis of the international competitions held show (Tezcan, 2013).

The decision making is one of the main heuristics in sports practice, it is defined as the process by which the athlete decides how to act or react according to the demands of the environment to achieve different performance objectives (Hodges et al., 2007); in short, it is about guessing the right information from the environment to plan future actions. In recent years, the study of the decision-making process in collective sports has received considerable attention; in football, it has studied the influence of contextual factors on decision-making by highly skilled players through semi-structured interviews, where the importance of considering the dynamic and static context in which players make decisions was demonstrated (Levi and Jackson, 2018); in volleyball, the relationship between decision-making and performance in three actions of the game (reception, scenario, attack) has been analyzed, through which, it was clear that the relevance of the decisional skills in the performance of the actions of the game, which contribute to the final performance of equipment (Conejero et al., 2018); a study made with players from NFL and NCAA about the theory of the risk sensitivity showed that athletes risk-sensitive decisions were made when the needs motivational interviewing were stronger (Gonzales et al., 2017); finally, in handball, players have been studied, showing the influence of game time for decision-making in relation to tactical media used (Prudente et al., 2017), and the referees, showing how the spatial situation of the same ones influences the success or error (Morillo-Baro et al., 2017b). And in beach handball, the influence of different contextual factors on decision-making by players in competition has been analyzed (Vázquez-Diz et al., 2019a).

Araújo et al. (2016) propose to study the process of decision making, based on the ecological perspective, observing the trace or behavioral traces of athletes through success patterns against unsuccessful patterns considering different factors such as space, teammates and rivals. The observation of this trace of behavior can be done through a mixed observation tool of Field Format and Category Systems E/ME (exhaustive and mutually exclusive) (Araújo et al., 2016). Systematic observation is a technique and/or methodology to analyze behavior in sports (Anguera and Hernández-Mendo, 2013) and several tools have been created to analyze what happens in the situations of game of handball (González, 2012; González et al., 2013; Sousa et al., 2014; Loffing et al., 2015; Helm et al., 2016; Jiménez-Salas and Hernández-Mendo, 2016) and beach handball (Morillo-Baro and Hernández-Mendo, 2015; Vázquez-Diz et al., 2019b).

Studies in the field of sport, in the framework of mixed methods, need to connect qualitative and quantitative elements (Plano-Clark and Sanders, 2015), and the ideal solution is to apply the successive steps defined within systematic observation, that is the main procedure to collect sport data in event analysis and there is ample experience with its use and evidence of its potential (Portell et al., 2015). Also, systematic observation is widely applicable and offers an optimal balance between rigor and flexibility. The observational records are qualitative, but the quantitizing (Tashakkori and Teddlie, 1998) is particularly robust, because apart from simple frequency counts, it contemplates other essential primary parameters, such as order and duration. This specific consideration of the order parameter is crucial for detecting hidden structures through the quantitative analysis of relationships between different codes in systematized observational datasets (Anguera et al., 2017). Just the wide scope of opportunities available for processing data derived from observation supports the idea that purely observational studies should be considered as mixed methods research studies, even though they constitute a somewhat special case and do not follow traditional patterns (Anguera et al., 2017).

Systematic observation is developed in natural or habitual contexts, and consists in a scientific procedure that, based on the considered objectives, reveals the occurrence of perceptible behaviors, to proceed to its registration organized by means of a specifically elaborated instrument and using the appropriate parameters. The motor behavior can be studied through the most characteristic analysis of the observational methodology, the sequential analysis of delays and the polar coordinate analysis (Anguera and Hernández-Mendo, 2013, 2014, 2015). The sequential analysis of delays is aimed at the detection of sequential patterns of behavior and is carried out by means of the search of sequential contingencies between categories or codes of behavior, through an observation instrument configured through a system of categories, field format or mixed instruments between category system and field format (Anguera et al., 2007).

Taking into account all this background on beach handball as an object of study, the aim of this research has been to observe the decision making of the specific position of the specialist through the observation of their actions. Identify, for each gender, the different significant associations between the behaviors of this specific position and those of the rest of the observation tool by analyzing polar coordinates.

## Materials and Methods

In the framework of mixed methods, the observational design used was nomothetic, punctual and multidimensional (Anguera et al., 2011). The unit of observation was the sequence of positional attack, which in this investigation will be defined from when the figure of the specialist appears in the attack until the change of possession occurs.

### Participants

Of the 24 teams that participated in the 2016 Senior Spanish Cup, six teams of each gender were selected to be observed (*M* ± SD: age male = 26.67 ± 5.85; age female = 23.27 ± 5.74). The six teams have been selected randomly. The research was conducted on a total of 24 observation sessions, twelve for the male category and twelve for the female category. The number of sessions estimated to generalize accurately (Morillo-Baro et al., 2015). Table 1 shows the matches that have been analyzed.

**Table 1:** Analyzed matches.

Category	Matches
Male	C. BMP. Alcala – Zonabalonmano Cadiz
	C. BMP. Ciudad de Malaga – C. BMP. Bahia de Algeciras (team no observed)
	C. BMP. Ciudad de Malaga – C. BMP. Barbate “B” (team no observed)
	BHC Plan B Barcelona – C. BMP. Barbate
	BHC Plan B Barcelona – Zonabalonmano Cadiz
	Pinturas Andalucia BMP Sevilla – C. BMP. Barbate
	Pinturas Andalucia BMP Sevilla – C. BMP. Alcala
Female	C. BMP. Algeciras – C. BMP. Ciudad de Malaga
	C. BMP. Algeciras – Deporte y Empresa Clinicas Rincon Malaga
	C. BMP. Ciudad de Malaga – C. BMP. Getasur juvenil Madrid
	C. BMP. Getasur junior Madrid – Deporte y Empresa Malaga
	C. BMP. Getasur junior Madrid – C. BMP. Getasur juvenil Madrid
	Jugui SOS Valencia (team no observed) – Deporte y Empresa Málaga
	Grupo Llopis BMP Sevilla (team no observed) – C. BMP. Algeciras

### Instruments

**Figure 1:**
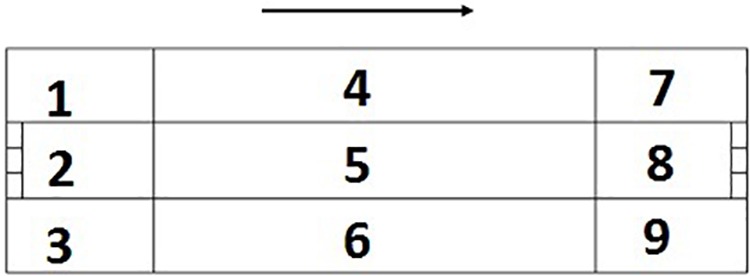
Division of game spaces. The numbering is related to the sense of attack.

The Royal Spanish Handball Federation (RFEBM) recorded the matches. The HOISAN v. 1.6.3.3.2 computer software was used (Hernández-Mendo et al., 2012) to perform the sequential analysis, the polar coordinate analysis and its vectorial representation, as well as the codification of the observations before to the sequential analysis. An *ad hoc* design tool has been used which has passed the quality tests of the data required in OM, (Morillo-Baro and Hernández-Mendo, 2015). It is built through a mixed system of field formats and exhaustive and mutually exclusive category systems (E/ME). The tool consists of 11 criteria and 90 categories that are complementary to the chronological development of an attack (Morillo-Baro and Hernández-Mendo, 2015). Table 2 shows the composition of the instrument of observation and Figure 1 the division of game spaces:

**Table 2:** Observation instrument: criteria and corresponding categories and codes.

Criterion	Categories	Criterion	Categories
1. Minute	M1: minute one of first half	2. Score	MPATE: tie
	M2: minute two of first half		1FAV: observed team is ahead by one point
	M3: minute three of first half		2FAV: observed team is ahead by two points
	M4: minute four of first half		M2FAV: observed team is ahead by more than two points
	M5: minute five of first half		1CON: observed team is trailing by one point
	M6: minute six of first half		2CON: observed team is trailing by two points
	M7: minute seven of first half		M2CON: observed team is trailing by more than two points
	M8: minute eight of first half		
	M9: minute nine of first half		
	M10: minute 10 of first half		
	M11: minute one of second half		
	M12: minute two of second half		
	M13: minute three of second half		
	M14: minute four of second half		
	M15: minute five of second half		
	M16: minute six of second half		
	M17: minute seven of second half		
	M18: minute eight of second half		
	M19: minute nine of second half		
	M20: minute 10 of second half		
	MGOL1: golden goal of first half		
	MGOL2: golden goal of second half		
3. Numerical balance	IGUAL: equality	4. Defensive system	S30: 3:0 defensive system
	1SUP: advantage of one		S21: 2:1 defensive system
	M1SUP: advantage of more than one		S2M1: 2 + 1 defensive system
	1INF: disadvantage of one		SPRES: individual defensive system
	M1INF: disadvantage of more than one		SREPL: retreat defensive system
			S20: 2:0 defensive system
5. Zone of end of attack	ZF1: attack ends in zone 1	6. Substitution area	BCSI: attack ends in one of the zones adjacent to the observed team’s substitution area
	ZF2: attack ends in zone 2		BCNO: attack ends in one of the zones adjacent to the opposing team’s substitution area.
	ZF3: attack ends in zone 3		BCMED: attack ends in one of the central zones.
	ZF4: attack ends in zone 4		
	ZF5: attack ends in zone 5		
	ZF6: attack ends in zone 6		
	ZF7: attack ends in zone 7		
	ZF8: attack ends in zone 8		
	ZF9: attack ends in zone 9		
7. Assisting player	APETO: assist by specialist	8. Player who ends attack	FPETO: specialist ends attack
	AXTRI: assist by left wing		FXTRI: left wing ends attack
	AXTRD: assist by right wing		FXTRD: right wing ends attack
	ACENT: assist by center		FCENT: center ends attack
	APIV: assist by pivot		FPIV: pivot ends attack
	NASIS: no assist		
9. How attack ends	FLY: attack ends with in-flight shot	10. Result of end of attack	GOL1: one-point goal
	GIRO: attack ends with spin shot		GOL2: two-point goal
	LANZ: attack ends with shot		ERLAN: missed shot
	6M: attack ends with 6-m throw		CPOSE: change of possession
	ERRPR: attack ends with passing or catching error		G1SA: one-point goal and suspension
	FTECN: attack ends with foul		G2SA: two-point goal and suspension
			6MGOL: goal scored by 6-m throw
			6MFA: missed 6-m throw
			GOL1R: one-point rebound goal
			GOL2R: two-point rebound goal
			6MGSA: goal scored by 6-m throw and suspension
			6MFSA: missed 6-m throw and suspension
			GOL1RSA: one-point rebound goal and suspension
			GOL2RSA: two-point rebound goal and suspension
11. Duration	D05: between 0 and 5 s				
	D610: between 6 and 10 s		
	D1115: between 11 and 15 s		
	D1620: between 16 and 20 s		
	D2125: between 21 and 25 s		
	D2630: between 26 and 30 s		
	DM30: more than 30 s		

### Procedure

#### Data Quality

Once the data collection is done, the observer must have the necessary guarantee about its quality (Anguera, 2003). Pearson’s, Spearman’s, Tau b of Kendall’s correlation coefficients and the Coefficient Phi have been evaluated. In addition, Cohen’s Kappa concordance index was calculated for the complete session, the results are shown in the Table 3.

**Table 3:** Intra and inter-observer agreement.

Coefficient for	Intra-observer	Inter-observer
entire session	agreement	agreement
	(Obs. 1 vs. Obs. 1bis)	(Obs. 1 vs. Obs. 2)
Pearson’s	0.98	0.97
Spearman’s (ρ)	0.95	0.89
Kendall’s tau-b	0.95	0.91
Kappa	0.94	0.91
Phi	0.89	0.85

Cohen’s Kappa index of this research exceeds the score of 0.90 for intra-observer (0.94) and inter-observer (0.91) reliability. Therefore, observing the values of Gelfand and Hartmann (1975) or Landis and Koch (1977), it can be considered that there is a high reliability.

#### Generalizability Analysis

For the application of the Theory of Generalizability, SAGT computer software has been used (Hernández-Mendo et al., 2016), This software has been used to determine the intra-observer and inter-observer concordance, through a design of two facets, categories and observers = C/O, in addition to assessing the homogeneity of the categories, but this time with a two-faceted design, likewise, but with an O/C design. The Theory of Generalizability unifies the definitions of reliability, validity and precision. The Generalizability study consists basically of four phases: (1) definition of the facets of study; (2) analysis of variance of the scores obtained on the study facets; (3) calculation of the error components; (4) optimization of the Generalizability coefficients (Blanco-Villaseñor et al., 2014).

A two-faceted design (categories and observers = C/O) has been established, as discussed above, to determine the reliability among observers (inter-observer concordance). The results shown by the report obtained through the SAGT program indicate that almost all the variability is associated with the facet categories (97.12%), being 0 for the facet observers and 2.28% for the interaction of the facets categories/observers. The coefficients of generalizability in this design structure determine results of 0.98, which means excellent results.

For the determination of intra-observer reliability, the same design has been taken. The results indicate that almost all the variability is associated with the facet categories (97.99%), being 0 for the facet observers and 2.01% for the interaction of the facets categories/observers. The coefficients of generalizability in this design structure determine results of 0.99, which means excellent results.

To assess the homogeneity of the categories, a two-faceted design has been used (observers and categories = O/C), this design allows to check the degree of differentiation of the proposed categories. The generalization coefficients for this design are invalid (0.000), this indicates that the homogeneity of the categories is optimal in the sense of differentiating.

#### Polar Coordinate Analysis

The polar coordinate analysis is an analysis technique widely used in recent years in the field of Sports Science is (Araújo et al., 2016; Maneiro et al., 2019; Vázquez-Diz et al., 2019a). There are numerous works that have been used to analyze different sports, football is the sport on that more research are published (Robles et al., 2014; Castañer et al., 2017; Maneiro and Amatria, 2018), although it can also be found in other sports such as taekwondo, basketball or karate (López-López et al., 2015; Nunes et al., 2015; Riveiro-Bozada et al., 2016). In handball, Sousa et al. (2014) characterized and detected effective behavioral vectors in offensive situations of two against two. And specifically, in the modality of beach handball, Morillo-Baro et al. (2015) analyzed the positional attack establishing differences by gender.

This technique has its origin in Sackett’s (1980) work and subsequent optimization with the “genuine technique” (Anguera, 1997), which allows to make a drastic reduction of data and a vectorial representation of the interrelationships established between the different categories that constitute the proposed taxonomic system (Hernández-Mendo and Anguera, 1999; Gorospe and Anguera, 2000). This technique is supported by a lag sequential analysis of prospective and retrospective delays (Sackett, 1980), with the genuine technique (Anguera, 1997) of the occurred successive conducts. The values obtained in the calculation of the conditioned probability will allow obtaining the parameter *Z*_sum_ (*Z*_sum_ = Σ*z*/√*n*, where n is the number of delays) (Cochran, 1954). The distribution of this parameter *Z*_sum_ has a $\bar{x}$ = 0 and a SD = 1.

In obtaining these values the interrelation map of behaviors or a map of polar coordinates can be built (Gorospe and Anguera, 2000). For the construction of behavioral maps, it is necessary to determine the vector modules (to be considered significant they must be equal to or greater than 1.96). The radius or length of the radius is calculated by the square root of the sum of the square of the *Z*_sum_ of the X (prospective) and the square of the *Z*_sum_ of the *Y* (perspective).

$$\text{Radius}\;\text{=}\;\sqrt{x^{2}\;+\;y^{2}}$$

The angle of the vector (φ) (which will depend on the quadrant where it is located) showing the nature of the relationship (Castellano and Hernández-Mendo, 2003). This angle (φ) is calculated as φ = Arc sine of *Y*/radius.

For coding and analysis of polar coordinates, the HOISAN computer program has been used (Hernández-Mendo et al., 2012). First, a sequential analysis is performed for each category of all the observations made with the selected criteria behavior, obtaining the Z results with a range of delays of -5 and 5. Once these values were obtained, the calculations were made to determine the *Z*_sum_ parameters (prospective and retrospective) (Sackett, 1980), the quadrant assignment, the radius, the angle, and the transformed angle (AT) for the rest of the categories (Castellano and Hernández-Mendo, 2003). The characterization of each quadrant is the following (Castellano and Hernández-Mendo, 2003):

Quadrant I [+, +]: The given behavior is motivated with respect to the pairing performance behavior in retrospective and prospective perspective.

Quadrant II [-, +]: The given behavior has a relation with respect to the pairing of motivation in retrospective perspective and of inhibition in prospective perspective.

Quadrant III [-,-]: The given behavior has a relation with respect to the pairing of inhibition in retrospective and prospective perspective.

Quadrant IV [+,-]: The given behavior has a relationship with the behavior of a motivated pairing performance in prospective perspective and inhibition in retrospective perspective.

The behavior selected as (focal) criteria have been: APETO and FPETO. These are the behaviors in which the role of the specialist is the main protagonist, in APETO (*peto* assists) the player who acts as a specialist makes the last pass to one of his/her teammates so that he/she finishes the positional attack by means of one of the ways of completion that are collected in the observation tool. While in FPETO (*peto* finalizes) is the specialist player who ends the action of the positional attack.

## Results

Therefore, the results obtained can be observed by analyzing polar coordinates for the selected behaviors as a criterion. Table 4 shows the mating behaviors that are significantly linked when the criterion behavior is APETO.The results show, by quadrant and category, that when the criterion behavior is APETO, in quadrant I, where it is augmented in respect to the pairing behavior in retrospective and prospective perspective, in the male category it is associated with four pairing behaviors, highlighting the zone of completion five (ZF5), the band of medium change (BCMED), and the result of a goal against (1CON); whereas in the female category it is associated with nine pairing behaviors, highlighting the throwing error (ERLAN), the flight (FLY), the player that finishes is the far right (FXTRD) for the finishing area nine (ZF9) and a defensive system two in line and one in advance (S21).

**Table 4:** Relationships between focal behavior APETO and conditional behaviors.

Criterion behavior	Q	Male			Female		
		Pairing behavior	Vector module	T.A.	Pairing behavior	Vector module	T.A.
APETO	I	FPETO	2.22	64.5	ERLAN	2.59	66.98
		ZF5	1.98	52.45	GOL1	2.82	4.88
		BCMED	2.61	31.49	IGUAL	2.92	14.99
		1CON	3.33	37.52	S21	2.92	51.19
					M2FAV	3.01	32.28
					FLY	3.45	45.86
					FXTRD	3.5	53.59
					ZF9	3.56	23.59
					2CON	5.19	70.36
	II	M2CON	4.31	168.58	D2125	2.8	128.52
		SPRES	2.21	124.66			
		LANZ	2.81	99.65			
	III	FXTRI	2.04	202.56	1INF	2	207.19
		6MGSA	2.09	236.98	ERRPR	2.23	224.14
		ZF4	2.37	191.17	1SUP	2.29	181.01
		D1620	2.57	232.89	S20	2.62	192.03
		S2M1	2.75	198.33	CPOSE	2.69	235.12
		M2FAV	2.77	208.08	2FAV	2.7	199.88
		GIRO	3.03	209.48	EMPATE	4.23	261.4
					D05	2.89	182
					ZF4	3.2	234.86
					1FAV	3.23	224.86
					SPRES	3.58	244.38
					1CON	3.77	229.08
	IV	FPIV	2.02	339.59	D610	2.78	320.55
		FLY	2.45	345.2			
		2CON	2.95	348.74			
		2FAV	3.21	271.17			
		6MGOL	3.33	352.09			

*Legend: Q, Quadrant; T.A., transformed angle; FPETO, specialist ends attack; ZF5, attack ends in zone 5; BCMED, attack ends in one of the central zones; 1CON, observed team is trailing by one point; ERLAN, missed shot; GOL1, one-point goal; IGUAL, equality; S21, 2:1 defensive system; FLY, attack ends with in-flight shot; FXTRD, right wing ends attack; ZF9, attack ends in zone 9; 2CON, observed team is trailing by two points; M2CON, observed team is trailing by more than two points; SPRES, individual defensive system; LANZ, attack ends with shot; D2125, between 21 and 25 s; FXTRI, left wing ends attack; 6MGSA, goal scored by 6-m throw and suspension; ZF4, attack ends in zone 4; D1620, between 16 and 20 s; S2M1, 2 + 1 defensive system; M2FAV, observed team is ahead by more than two points; GIRO, attack ends with spin shot; 1INF, disadvantage of one; ERRPR, attack ends with passing or catching error; 1SUP, advantage of one; S20, 2:0 defensive system; CPOSE, change possession; 2FAV, observed team is ahead by two points; EMPATE, tie; D05, between 0 and 5 s; 1FAV, observed team is ahead by one point; FPIV, pivot ends attack; 6MGOL, goal scored by 6-m throw; D610, between 6 and 10 s*.

In quadrant II, where the criterion behavior has a relationship with respect to that of augmented performance in pairing in retrospective perspective and of inhibition in prospective perspective, in male category, it is only associated with three pairing behaviors, being the result more than two in against (M2CON) the one that greater intensity in the relationship presents. Meanwhile, in the female category, only a pairing behavior appears, which is the duration of the attack between 21 and 25 s (D2125).

In quadrant III, the criterion behavior has a relationship with respect to the pairing of inhibition in retrospective and prospective perspective. In the male category, there are seven pairing behaviors, of those it can stand out the way of ending in spin shot (GIRO), the defensive system of the opposing team two plus one (S2M1), the player that ends is the left wing (FXTRI), the zone of end four (ZF4) and the result of more than two goals in favor (M2FAV). In the female category, it is related to twelve pairing behaviors, of which can distinguish the defensive system of the opposing team (SPRES), the pass or reception error (ERRPR), the change of possession (CPOS) and the duration of the attack less than 5 s (D05).

Finally, in quadrant IV, the criterion behavior has a relationship with the behavior of augmented pairing in prospective perspective and inhibition in retrospective perspective. In masculine category, it is related to five pairing behaviors, being the result of two goals in favor (2FAV) the behavior that greater radio has shown, while, in feminine category, it is associated only with a pairing behavior, the duration of the attack between 6 and 10 s (D610).

**Figure 2:**
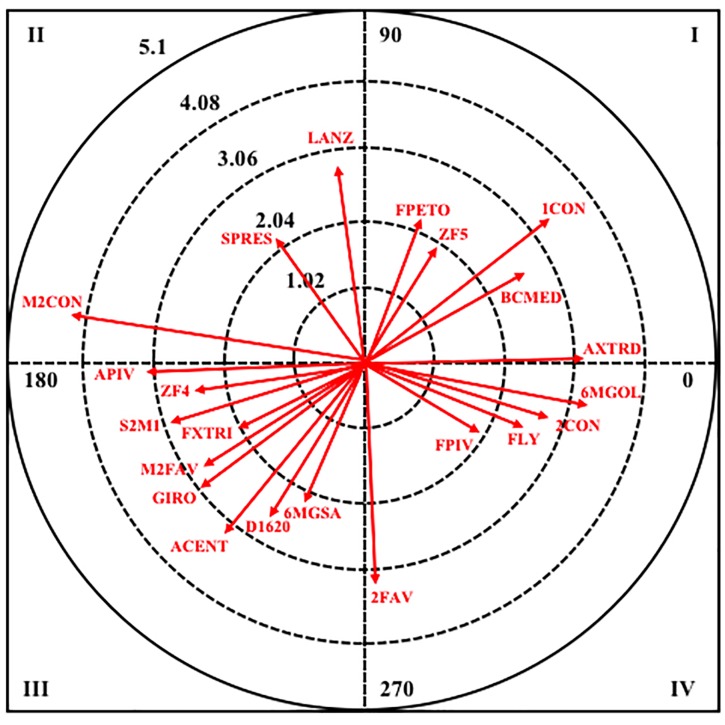
Vector map for focal behavior APETO in male category.

Next, in Figure 2, it can be observed the resulting graphical representation from the analysis of polar coordinates for this criterion behavior in male category.

**Figure 3:**
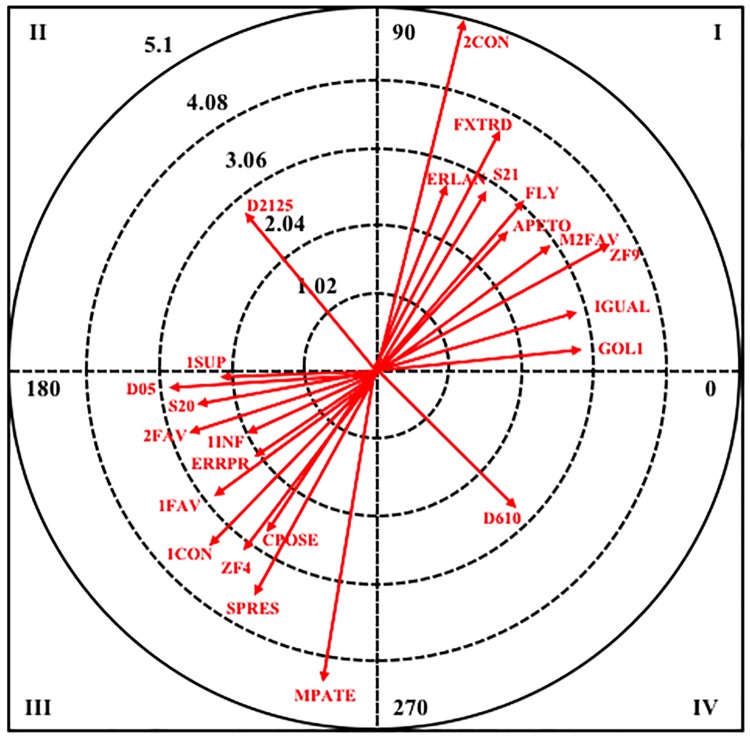
Vector maps for focal behavior APETO in female category.

In Figure 3, is represented the graphical representation for the female category.

Table 5 shows the results obtained when the criterion behavior is FPETO.

**Table 5:** Relationships between focal behavior FPETO and conditional behaviors.

Criterion behavior	Q	Male			Female		
		Pairing behavior	Vector module	T.A.	Pairing behavior	Vector module	T.A.
FPETO	I	ZF5	2.17	34.71	ZF4	2.28	32.94
		LANZ	2.74	34.58	S2M1	2.54	71.6
		BCSI	3.34	52.87	MPATE	2.84	35.19
		ZF6	3.48	78.49	2FAV	3.99	53.07
		1FAV	3.97	8.29			
		1CON	5	48.87			
		S30	5.39	28.5			
	II	2CON	2.3	148.77	D610	2.04	149.28
		D610	1.98	216.47	ZF7	2.46	197.64
		MPATE	2.06	191.46	M2FAV	3.8	208.53
		2FAV	2.27	265.28			
		ZF8	2.29	227.09			
	III	6MGSA	2.32	191.5			
		ERLAN	2.39	188.04			
		M2FAV	2.92	219.93			
		FLY	3.42	240.37			
		BCNO	3.49	214.29			
		AXTRD	3.7	212.82			
		S21	6.72	215.09			
	IV	ZF9	2.42	308.21	2CON	2.13	273.57
					6M	2.16	334.60
					DM30	2.41	343.01
					D05	2.51	341.55

*Legend: Q, Quadrant; T.A., transformed angle; ZF5, attack ends in zone 5; LANZ, attack ends with shot; BCSI, attack ends in one of the zones adjacent to the observed team’s substitution area; ZF6, attack ends in zone 6; 1FAV, observed team is ahead by one point; 1CON, observed team is trailing by one point; S30, 3:0 defensive system; ZF4, attack ends in zone 4; S2M1, 2 + 1 defensive system; MPATE, tie; 2FAV, observed team is ahead by two points; 2CON, observed team is trailing by two points, D610, between 6 and 10 s; ZF8, attack ends in zone 8; ZF7, attack ends in zone 7; M2FAV, observed team is more than two points; 6MGSA, goal scored by 6-m throw and suspension; ERLAN, missed shot; FLY, attack ends with in-flight shot; BCNO, attack ends in one of the zones adjacent to the opposing team’s substitution area; AXTRD, assist by right wing; S21, 2:1 defensive system; ZF7, attack ends in zone 7; ZF9, attack ends in zone 9; 2CON, observed team is trailing by two points; 6M, attack ends with 6-m throw; DM30, more than 30 s; D05, between 0 and 5 s*.

In quadrant I, in male category, it is related to seven pairing behaviors, being the most important the finishing zone five (ZF5) and six (ZF6), the finishing mode in launch (LANZ), the defensive system of the opposing team 3:0 (S30), and the result of a goal in favor (1FAV) and a goal against (1CON). In female category, on the other hand, it can stand out the finishing zone four (ZF4) and the defensive system two in line plus one player in advanced individual defense.

In quadrant II, in the male category, a pairing behavior appears only as the result of two goals against (2CON), as in the female category, the duration of the attack between 6 and 10 s (D610).

In quadrant III, in the male category, it is related to eleven pairing behaviors, highlighting the missed shot (ERLAN) and the defensive system of the opposing team, two players in line and one advanced (S21), this being the one with the largest module of vector. In the female category, only two pairing behaviors appear, the result of more than two goals ahead (M2FAV), which is the one with the highest radius, and the zone of completion seven (ZF7).

In quadrant IV, in the male category only one pairing behavior is found, the zone of completion nine (ZF9); while in the female category four can be found, emphasizing the duration of the attack of 5 s (D05) over the rest.

**Figure 4:**
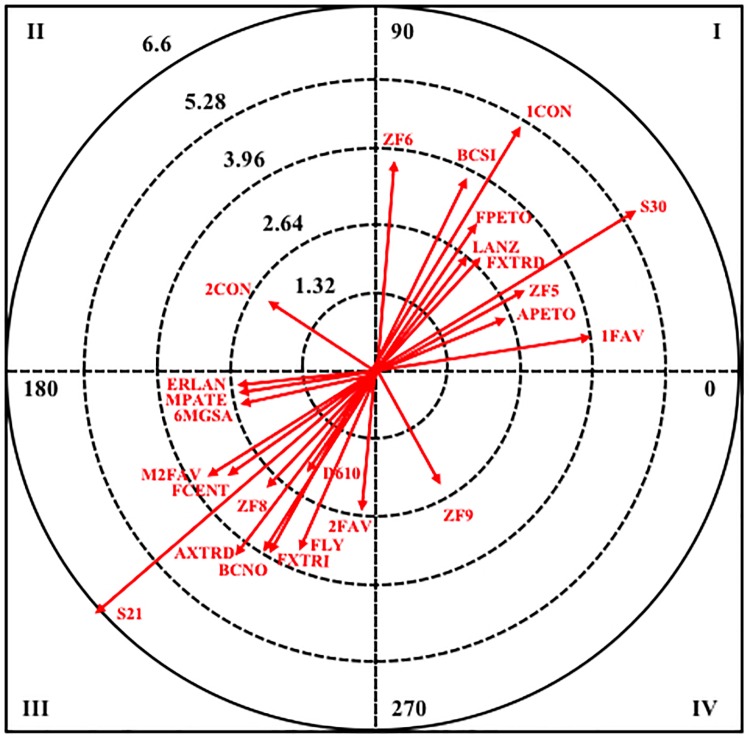
Vector maps for focal behavior FPETO in male category.

Next, in Figure 4, the graphical representation resulting from the analysis of polar coordinates for this focal behavior can be observed.

**Figure 5:**
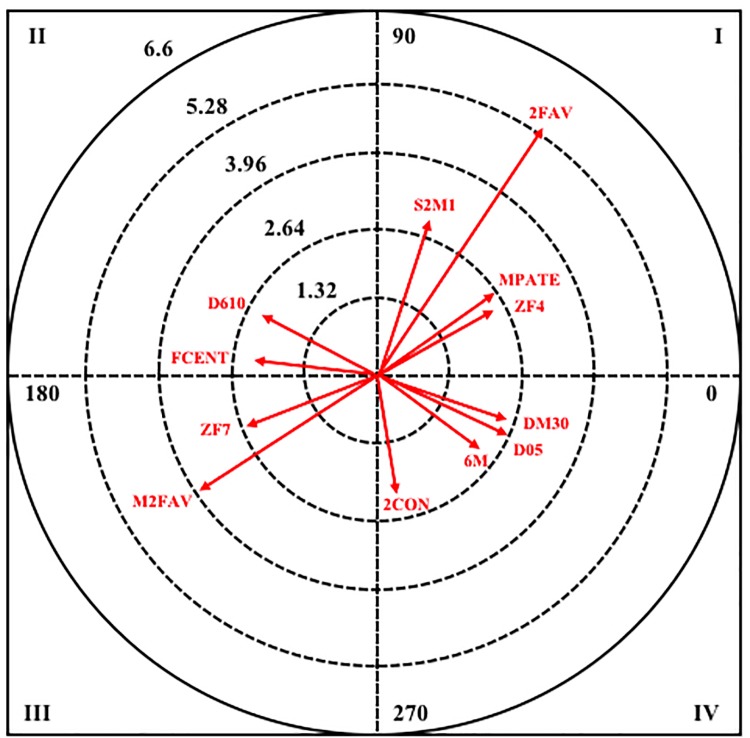
Vector maps for focal behavior FPETO in female category.

In Figure 5 the graphical representation for the female category is represented.

## Discussion

The aim of this study was to analyze the decision making of beach handball players who acquire the role of specialist according to the behavior they have shown, from the framework of mixed methods. To do this, the aim has been to identify the relationships that were established between behaviors used as a criterion, those that showed their decision-making by performing assists and completions, with pairing behaviors of the selected criteria of the tool: score, defensive system of the opposing team, zone of end of the attack, player that ends, end mode and duration of the attack. The results showed differences between the flow of behaviors that are significantly linked in the male category compared to the female category, which coincides with the results obtained in the studies published on this modality so far (Morillo-Baro et al., 2015; Vázquez-Diz et al., 2019a).

Firstly, and in the male category, the specialist’s assists are associated with the score against, which is manifested in the four quadrants of the table of results. These indicate that the situation of numerical superiority and his ability to fix opponents do not positively relate the score with the performance of assists, therefore, it seems that the participation of this position goes more toward decisions of continuity in actions and completion of the attack than toward decision making of the last pass. In this sense, the research by Vázquez-Diz et al. (2019a) relates the success in the decision making in a positive way with a favorable score. Another remarkable result is that the area of the court where male teams tend to end their attacks when the assist is made by the specialist is in the central zone, results that do not coincide with those obtained by Morillo-Baro et al. (2015). Data that shows a tactical evolution in the game in male category, either to solve the absence of left-handed players or to look for solutions before the increasingly better defensive systems. In addition, the assist of the specialist player cannot be associated with the completion in turn, a result that falls within the logic, since this gesture does not need tactical collaboration unlike the shot in flight (Morillo-Baro et al., 2017a). When inhibiting the behaviors referring to the defensive pressing systems, it is reasonable to think that this player performs his actions before closed defensive systems, a result that agrees with the research of Morillo-Baro et al. (2015). Individual defensive systems have a negative performance improvement in the women’s category, specifically with errors of pass and reception, as shown, the results of the study by Vázquez-Diz et al. (2019a).

On the other hand, in relation to the specialist’s completion in the male category, both the score for and against the central and right end zone and by a launch faced with a closed defensive system can be associated, as behaviors are inhibited related to open and pressing defenses; data that agree in part with those of the Morillo-Baro et al. (2015), since in that research the closed defensive system and the favorable score are also augmented. A result that could be due to the entrance of the specialist player in his most effective shooting area and, being the majority of the players that act in this position of right-handed players, to the natural tendency to play toward the players strong point, where he has more guarantees to shoot. Finally, when he decides to finalize he does it successfully, because shoot errors are inhibited and they are not linked to other possible negative completion options for the team, such as technical fouls or pass-reception errors, aspects that show his correct choice in the game of numerical superiority.

For the female category, the specialist’s assist activates the completion by the extreme right in flight, in her usual area of action and before an open defensive system. This is a result that could be due to the fact that the majority of players who occupy the position of specialist are right-handed, and therefore they tend to play toward their strong point, from left to right. The open defensive systems chosen by the women’s teams are based on the need to limit the specialist’s actions, since their participation in decision making and completion is more decisive than in the male category (Morillo-Baro et al., 2015). It is worth mentioning that, although the error in shooting is also activated, it should not be interpreted as an erroneous decision of the specialist since, although the right end fails the shot, the attack always ends with the option to score. Reasoning that is reinforced with the inhibition of pass-reception error behavior and change of possession in quadrant III; the inhibition of these behaviors, related to the failure of the positional attack, could be associated with a correct decision making by the specialist. The results of this quadrant, like those of the fourth, also indicate that the specialist’s assist is usually given once the first 10 s have passed.

Regarding the specialist’s finalization, the fact that it occurs toward the left end zone stands out, toward her weak point if it is a right-handed player and faced with an open defensive system. And comparing the duration of the attack when she decides to finish instead of assisting, the players elaborate the attack less, developing it in less time. Contrary to the results shown by Vázquez-Diz et al. (2019a) where a longer length of attack is related to behaviors of success.

Based on all these results, one might think that in the male category the decision of the specialist’s decision-making when he assists is linked to an unfavorable score, while in the female category that decision making is independent of the score. On the other hand, when the specialist decides to finish it is interpreted as a correct decision making since it inhibits behaviors related to the failure, while, in the feminine category, there are no behaviors to link to the result of the completion, therefore, it can mean success or not in the decision making. It is also noteworthy that in the male category both the completion and assistance of the specialist is given defensive closed system, a logical issue that allows greater freedom of action on the part of this player, while in the female category despite having open defensive systems they do not decrease the actions of this player, data that reveals the importance of this role, more if possible, in this category. Finally, the specialist’s assist is not related to the turn in the male category and in the female category it is related to the flight, this means that both categories use the flight as a main resource against the turn, data that do not agree with those obtained by Morillo-Baro et al. (2015) and Ostoić and Ohnjec (2015), where the turn proved to be the main resource to obtain double scoring goals in both categories.

There is hardly any published research on this modality, which suggests some caution to draw conclusions about the estimated data. One of the cautions is the limitation that means to interpret decision making by observing behaviors, being unaware of other cognitive variables that surely influence. Finally, it is also important to note that the sample was composed only by Spanish players; therefore, this study is focused solely on the development of the game of Spanish teams. All these results have shown the importance of the specific position of the specialist in the development of the attacks within the offensive systems of the teams, and the greater or lesser accuracy that they have when the decision making of this player is correct or not. The use of systematic observation to analyse specific actions and behaviors, in the framework of mixed methods, can provide complementary ideas on the complex process of decision making in sport (Araújo et al., 2016). This has been possible thanks to the use of polar coordinate analysis, a tool that has become very useful to detect the significant relationships of the criterion behaviors chosen with the different pairing behaviors that correspond to the different facets of the game. For all this, it would be of great interest to continue exploring this line of research on beach handball, creating new investigations with different focal behaviors to be able to establish the different patterns of the game in both categories.

## Author Contributions

AH-M, VM-S, and RR designed the study and acquired, analyzed, and interpreted the data. JM-B acquired, analyzed, and interpreted the data. JV-D acquired and analyzed the data. All authors drafted, revised, and approved the final version of the manuscript, and agreed to be accountable for all aspects of the work.

## Conflict of Interest Statement

The authors declare that the research was conducted in the absence of any commercial or financial relationships that could be construed as a potential conflict of interest.
